# Functioning free gracilis transfer to reconstruct elbow flexion and quality of life in global brachial plexus injured patients

**DOI:** 10.1038/srep22479

**Published:** 2016-03-03

**Authors:** Yi Yang, Jian-Tao Yang, Guo Fu, Xiang-Ming Li, Ben-Gang Qin, Yi Hou, Jian Qi, Ping Li, Xiao-Lin Liu, Li-Qiang Gu

**Affiliations:** 1 Department of Microsurgery and Orthopedic Trauma, the First Affiliated Hospital of Sun Yat-sen University, Guangzhou 510080, China; 2 Department of Orthopedic Surgery, the First Affiliated Hospital of Henan University of Science and Technology, Luoyang 471003, China

## Abstract

In the study, the functional recovery and relative comprehensive quality of life of cases of global brachial plexus treated with free functioning muscle transfers were investigated. Patients who received functioning gracilis muscle transfer between August 1999 and October 2014 to reconstruct elbow flexion, wrist and fingers extension were recruited. The mean age of the patients was 26.36 (range, 16–42) years. The mean period of time from gracilis transfer to the last follow-up was 54.5 months (range, 12–185 months). Muscle power, active range of motion of the elbow flexion, wrist extension, and total active fingers extension were recorded. SDS, SAS and DASH questionnaires were given to estimate patients’ quality of life. 35.71% reported good elbow flexion and 50.00% reported excellent elbow flexion. The average ROM of the elbow flexion was 106.5° (range, 0–142°) and was 17.00° (range, 0–72°) for wrist extension. The average DASH score was 51.14 (range, 17.5–90.8). The prevalence of anxiety and depression were 42.86% and 45.24%. Thrombosis and bowstringing were the most common short and long-term complications. Based on these findings, free gracilis transfer using accessory nerve as donor nerve is a satisfactory treatment to reconstruct the elbow flexion and wrist extension in global-brachial-plexus-injured patients.

Traumatic brachial plexus injury (BPI) is a devastating lesion that causes severe upper extremity disability, especially in those patients who suffer from global BPI. Unfortunately, there is a lack of effective therapeutic approaches for this injury.

Global BPI can be treated within one year with nerve transfer procedures such as the contralateral C7 (CC7), phrenic, spinal accessory, intercostal nerve, and motor nerve of the cervical plexus^1^.

In cases with unsatisfactory outcome of primary nerve reconstruction, or denervation period more than one year, functioning free muscle transfer (FFMT) is a reliable option to reconstruct the disabled limb function.

FFMT was first introduced for facial reanimation^2^ or Volkmann’s contractures^3^. Since FFMT was introduced for elbow flexion in BPI reconstruction^4^, different muscles have been proposed to reconstruct upper extremity function after BPI; the gracilis remains the most widely used by different doctors^5^,^6^,^7^.

Quality of life (QOL) in BPI patients after reconstructive surgery, with or without free muscle transfer, has been reported^8^,^9^,^10^,^11^ by using disabilities of the arm shoulder and hand (DASH) and 36-item short-form (SF-36), while the psychological state of BPI patients have not been described yet. Because mental health may play an important role in the long-term effect of QOL, it is important to pay attention to the patients’ psychological problems.

The purpose of the present study was to investigate the functional recovery and relatively comprehensive QOL in global BPI patients after FFMT.

## Materials and Methods

### Patients

The preliminary diagnosis of BPI was based on detailed histories, physical examinations, electromyography (EMG) and MRI. The inclusion criteria were patients who received functioning gracilis muscle transfer (FGMT) to reconstruct elbow flexion, wrist extension, and fingers extension between August 1999 and October 2014 in our hospital. The exclusion criteria were patients whose follow-up had been less than 12 months and those who received an additional procedure to reinforce elbow flexion, wrist extension, and fingers extension. Age, gender, etiology, complications, follow-up times, and the outcomes were respectively recorded. All surgeries and perioperative management were done by the same surgeon (Li-Qiang Gu) and his medical team.

### Surgical procedures

With the patient in the supine position, the operation was carried out under general anesthesia. The gracilis with the surrounding fascia preserved was harvested with the anterior branch of the obturator nerve, together with its vascular supply, which is a branch of the profunda femoris artery; a skin paddle was typically harvested to facilitate postoperative flap monitoring as well. The entire gracilis, with tendon from pubic symphysis to the pes anserine insertion distal, was taken and positioned in the recipient site so that the nerve was as close as possible to the donor nerve.

The gracilis was placed subcutaneously in the anterio-medialis aspect of the arm, passed under the pulley formed by the brachioradialis, extensor carpi radialis longus and extensor carpi radialis brevis muscle. The proximal part of the gracilis muscle was sutured to the acromion or the distal portion of the clavicle; the distal part was sutured to the extensor digitorum communis tendon and extensor pollicis longus tendon with double interlacing sutures.

**Figure 1:**
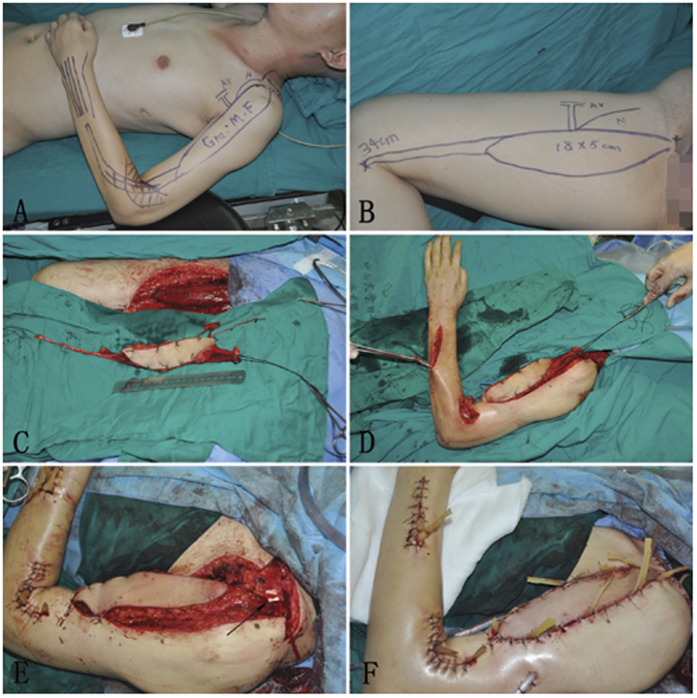
(**A**) Surgical design of the recipient site before operation; (**B**) Surgical design of the donor site before operation; (**C**) Showed the gracilis was dissected from the thigh; (**D**) The gracilis was placed subcutaneously in the anterio-medialis aspect of the arm; (**E**) The nerve of the gracilis was anastomosised to the accessory nerve (arrow); (**F**) Rubber sheets and tube were used for drainage after surgery.

The muscle was perfused by the brachial artery, axillary artery, subclavian artery, etc. with T-shaped or end-to-end anastomosis^12^ and refluxed by comitant vein, such as cephalic vein, brachial vein, and axillary vein. The nerve to the gracilis was coapted to the spinal accessory nerve (SAN) (forty-five patients) and phrenic nerve (two patients) using 9-0 nylon sutures with the assistance of a 10 power surgical microscope (Fig. 1). The transferred muscle was returned to its resting length after suturing.

### Post-operative care

The patient was required to wear a cast with elbow flexion of 90°, wrist in neutral position and fingers full extension for 6 weeks after the surgery.

Patients were required to do rehabilitation and electrical stimulation therapy regularly and to take neurotrophic drugs, as well as being followed up postoperatively on a regular basis.

The muscle power was recorded using modified MRC standard and the active range of motion (ROM) of the elbow flexion, wrist extension, and total active fingers extension (TAFE).

### Questionnaires

The patients were given the DASH, Self-Rating Depression Scale (SDS), and Self-Rating Anxiety Scale (SAS) questionnaires (all in Chinese version) to estimate their QOL and the Numeric Rating Scale (NRS) was used to evaluate the pain. The ethics committee of the First Affiliated Hospital of Sun Yat-sen University approved the study, and all patients gave written informed consent. All methods were carried out in “accordance” with the approved guidelines.

The DASH questionnaire has been validated for measuring upper limb function. This questionnaire is composed of 30 questions regarding patients’ symptoms and their ability to perform certain activities. Each question is scored on a scale from 1 to 5, with a minimum of 0 (no disability) and a maximum total score of 100(the severest disability)-a lower score represents better results while a higher score reflects a greater degree of disability.

NRS represents the pain level on a scale from 0 to 10. (0 represents there is no pain; 1 to 3, mild pain; 4 to 6, moderate pain; and 7 to 10, severe pain).

The SDS and SAS were used to estimate the patients’ depression and anxiety states. The Chinese versions of SAS and SDS scales have been confirmed reliable and validated by previous investigation^13^. Each questionnaire is composed of 20 items. Each item is scored on a scale of 1–4 (never, some of the time, relatively often, most of the time). The cutoff point of SAS scores ≥50 and SDS scores ≥53 reflected the existence of anxiety and depression, respectively. The SDS or SAS scores were classified into four categories of depression or anxiety severity: For the SDS scores, normal (below 53 points), presence of minimal to mild depression (53–62 points), presence of moderate to marked depression (62–72 points) and presence of severe to extreme depression (72 points and above); For the SAS scores, normal (below 50 points), presence of mild to moderate anxiety levels (50–59 points), severe anxiety levels (59–70 points) and presence of extreme depression (70 points and above).

### Statistical analysis

Continuous variables were presented as mean ± standard deviation (SD) and were compared by means of independent sample t-test or ANOVA, when normal distribution is satisfied, otherwise it was expressed as median and quartile range and the difference between groups were compared by nonparametric tests. Ordinal categorical variables were expressed as frequency and were compared by Wilcoxon rank sum test or Kruskal-Wallis H test. A two-tailed p < 0.05 was considered statistically significant. All analyses were performed using SPSS Version 20 (SPSS Statistics V20, IBM Corporation, Somers, New York).

## Results

From August 1999 to October 2014, 47 patients who had received a total of 49 gracilis transfers were included in this study. Two patients suffered from thrombosis and received a second gracilis transfer immediately. Five patients who were lost at the final follow-up were excluded from this study.

The most common gender, sides, and cause of injury were male (95.24%), left side (69.05%), and motorcycle accidents (76.19%), respectively. The mean age of the patients was 26.36 (range, 16–42) years while the mean period of time from gracilis transfer, to the last follow-up, was 54.5 months (range, 12–185 months) (Table 1).

**Figure 2:**
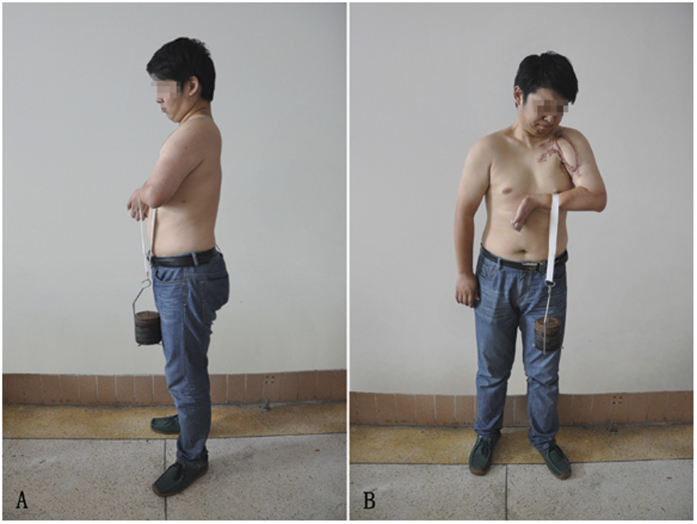
This patient came to our hospital with a global BPI after a motorcycle accident and primary nerve surgery failed. Transfer of gracilis reinnervated with accessory nerve. He can lifted a 5kg weight at the final follow-up (63 months). (**A**) Lateral view (**B**). Frontal view.

**Figure 3:**
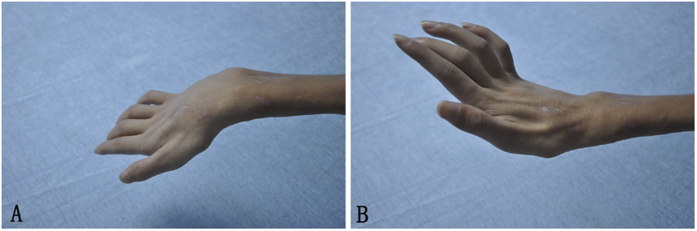
Patient recover satisfactory wrist and fingers extension after gracilis transfer. (**A**) Natural state (**B**). Active wrist and fingers extension state.

A grade of M0 to M2 constituted a poor result; M2 + to M3 was fair; M3 + to M4−, good; and M4 or M4 + , excellent. The results presented as good and excellent for elbow flexion, respectively, were 35.71% and 50.00% (Fig. 2). For wrist extension, good and excellent results were 14.29% and 7.14%, respectively. The average ROM of the elbow flexion was 106.5°(range, 0–142°) and the average ROM of wrist extension was 17.00°(range, 0–72°) (Table 2 and Fig. 3) . Based on the first contraction of the transferred gracilis, the mean reinnervation time was 5.8 ± 1.8 months (range, 3–12 months).

The percentages of no pain, mild, moderate and severe pain were 21.43%, 28.57%, 30.95%, and 19.05% respectively. The average DASH score was 51.14 (range, 17.5–90.8).

The prevalence of anxiety and depression were 42.86% and 45.24%, respectively. 35.71% of patients had both anxiety and depression. There were 9.52% of patients who suffered from severe depression and 2.38% suffered from severe anxiety. (Table 3)

No statistically significance was found between sides, ages, follow-up times, BMI, and those patients with or without bowstring complications or donor site complications in the measurements related to elbow flexion and wrist extension muscle power or ROM (Table 4 and Table 5).

The main complications in this study were divided into short- and long-term ones.

Short-term complications included four thrombosis (two arterial thromboses, two venous thromboses), three occurrences of gracilis partial necrosis (one proximal, two distal), two fat liquefaction, three hematoma (two donor site, one recipient site). Five patients suffered from numbness corresponding to the cutaneous territory of the obturator nerve while one patient suffered from numbness and reduction of adduction strength simultaneously.

**Figure 4:**
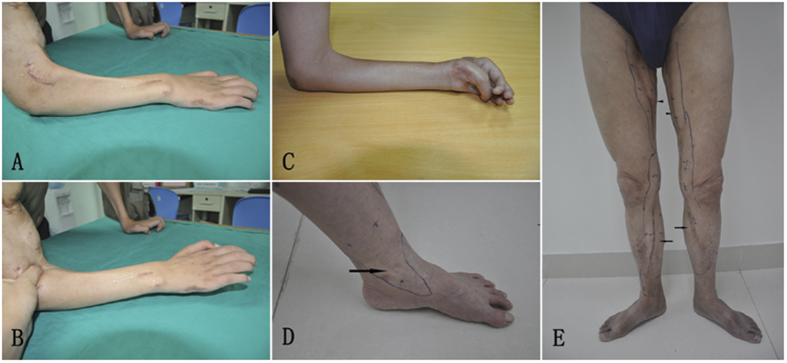
Long-term complications: (**A**) Showed the bowstrings complication. (**B**) The wrist and fingers extension improved after compress the “pulley site”. (**C**) This patient suffered severe stiffness of the elbow, wrist and fingers which lead to barely joints movement. (**D**) Showed the mild edema of ankle joint (arrow). (**E**) Showed the patient suffered hypoesthesia at the donor site; arrow head showed the hypoesthesia zone after gracilis dissection, arrow showed the hypoesthesia zone after initial sural nerve dissection.

Long-term complications included eight patients who suffered from bowstringing at the elbow and one patient who suffered from elbow stiffness. Three patients had numbness and one patient had hypoesthesia at the donor site. One patient reported knee instability but no objective medial laxities were found upon examination. One patient suffered from mild edema of ankle joint (Table 6 and Fig. 4).

## Discussion

Traumatic global BPI affects patients in their prime of life with significant function loss, which can cause severe socioeconomic consequences^14^,^15^.

Terzis *et al.*^16^ reported that the philosophy for upper limb reconstruction is from proximal to distal joints while Kay^17^ *et al.* reported that the primary goal is to restore elbow function. Elzinga^18^ *et al.* reported the elbow flexion is the leading aim of upper limb reconstruction followed by shoulder stability and wrist and hand function. The hand has the farthest distance compared with the shoulder, elbow and wrist; therefore it needs the longest time to recover after injury. Since the hand has the most complicated intrinsic muscles, it is very difficult to restore function. If patients had shoulder, elbow and wrist reconstruction first, it would prolong the time. As a result, it will lead to hand muscle atrophy that causes insufficient function recovery. Therefore, the principle in our hospital is to use nerve transfer to reconstruct the shoulder abduction and fingers flexion followed by reconstruction of elbow flexion and wrist and finger extension by functioning free gracilis transfer. The phrenic nerve was transferred to the suprascapular nerve to restore shoulder abduction and CC7 was commonly used to restore finger flexion in the first stage. Tu *et al.*^19^ transferred CC7 to the musculocutaneous nerve, 65% patients obtained at least M3 motor recovery of hand grip function. Wang *et al.*^20^ transferred CC7 to lower trunk, 64% and 53% patients attained finger flexion and thumb flexion (the muscle power were M3 + or greater), respectively. We had similar outcomes compared to these studies. For those patients who did not restore satisfactory finger flexion after nerve transfer, will receive a second FFMT for finger flexion as described by Doi. This study was focus on the outcome of the first FFMT to reconstruct elbow flexion and wrist and finger extension. The outcomes of the nerve transfer and second FFMT will be reported in the future. Many factors are considered to have an impact on the FFMT outcome, such as the shortest reinnervation time and distance, decrease of the nerve fiber loss, no nerve graft, tension-free nerve coaptation, access to nerve and reliability, especially in selecting the appropriate donor nerve^21^. All the extraplexus or intraplexus donor nerves can be used for FFMT^22^. In global BPI cases the extraplexus donor nerves include the SAN^23^, the IC^24^, phrenic nerve^25^ and the CC7^26^. Although some scholars reported insufficient outcomes using the SAN as donor in BPI reconstruction^17^,^27^ leads those surgeons not to choose the SAN as a priority donor nerve for the FFMT. Due to the easy dissection, mild donor site morbidity, earlier function recovery and the satisfied ultimate muscle power, the SAN is still the most commonly used donor nerve for FFMT neurotization in BPI reconstruction in our hospital. In this study, the muscle powers were good (35.71%) or excellent (50.00%) for elbow flexion. The consistency with other results^23^,^28^ proved that the SAN is effective as donor nerve. In addition, Gutowski^29^ *et al.* reported there were 1700 myelinated axons and Bhandari *et al.*^30^ reported the number of myelinated axons were 1,671 in the SAN, which indicated that spinal accessory nerve could be used as a satisfactory donor nerve. In two patients for whom the SAN is injured because of the trauma, we used the phrenic nerve as a donor nerve; they gained 4^−^ or 4 muscle power for elbow flexion. Although there were not enough cases in this study, the finding still represented that the phrenic nerve might be an effective choice when the SAN is not available. The SAN and phrenic nerves were made of pure motor nerve fibers which may restore better function than the intercostal nerves; besides, the proximal part of the gracilis muscle was sutured to the acromion or the distal portion of the clavicle, the SAN and phrenic nerve could anastomosis to the nerve of gracilis more conveniently.

Although some scholars prefer two years after surgery as the follow up time, this study shows that there is no significant difference between the two groups when comparing the muscle power and ROM. This suggests that after one year of normalized rehabilitation, patients can recover gracilis muscle power, which can contribute to elbow flexion, wrist extension, and digital extension. Furthermore, those patients who had poor function recovery, showed no improvement after two years or more rehabilitation in this study.

The most common complications in other reports included wound infections, delayed healing, and unsightly scarring^31^. In this study, donor site numbness and bowstringing were the most common short and long-term complication. Five patients had numbness after the surgery but two patients recovered one year later. As the most serious short-term complications postoperatively, thrombosis is the most predominant reason that affects the transferred muscle survival. Early appropriate intervention might be the only choice to save the transferred muscle. In this study, two patients gained excellent muscle power at the final follow-up, while another two patients gained unsatisfactory outcome. For those three patients who suffered from partial gracilis necrosis, after early debridement, the transferred muscle survives. They all gained satisfactory muscle power at the final follow-up. Partial gracilis necrosis is due to the muscle flap design beyond the gracilis muscle artery perfusion. The gracilis has two vascular patterns classically, both a dominant pedicle and minor vascular pedicles^29^. The entire gracilis could be nourished by the dominant pedicle. However, when the surgeon encounters a large gracilis, in order to avoid the partial gracilis necrosis, a vascularity assessment of the muscle before transplantation is necessary. According to our experience, early appropriate intervention could avoid thrombosis and the partial gracilis necrosis that lead to final gracilis transfer failure.

Bowstringing of the transferred gracilis may lead to decreased muscle power and ROM for the elbow flexion, wrist and fingers extension. In this study, eight patients suffered bowstringing of the gracilis muscle transfer at the elbow when the brachioradialis, extensor carpi radialis longus and extensor carpi radialis brevis muscle acted as pulley. But interestingly, the muscle power and ROM showed no statistical significance between patients with or without this complication; this might be due to limited cases. Barrie *et al.*^23^ described improved outcomes by using the flexor carpi ulnaris and Kate Elzinga *et al.*^18^ reported the distal portion of the flexor carpi ulnaris and palmaris longus at the level of the proximal forearm to create a more effective pulley, but still these modified procedures could not eliminate the bowstring, which leads to the transferred muscle’s invalid excursion. During the follow-up, we asked those patients about the bowstringing complication, patients felt that the muscle power and ROM were enough for daily life. So we did not perform supplement procedure to deal with the bowstringing complication. To resolve this bowstringing problem, we believe a more effective pulley is needed. Doi *et al.*^32^reported 10 muscle transfers in 19 patients needed tenolysis, while none of the patients in another group who receive modified rehabilitation exercises needed tenolysis after the gracilis muscle transfer surgery. To avoid adhesion to the surrounding tissues, the gracilis was harvested with the fascia preserved and patients were advised to do rehabilitation exercises in this study as described before. One of those patients suffered from severe elbow stiffness because of adhesion. Although the incidence is relatively low, this complication still needs attention because severe elbow stiffness can lead to poor outcome, which approximates to surgery failure.

We also compared the muscle power and ROM between patients with and without donor site complications. No significant difference was observed, illustrating that with appropriate intervention, a certain degree of complications may not affect the free gracilis transfer result.

There are several studies using DASH to evaluate global BPI patients’ functional outcomes and QOL^9^,^33^. The DASH scores (51.14 ± 20.97) in this study were better than some previous reported outcomes in traumatic global BPI at the final follow-up. Ahmed-Labib *et al.* reported a mean DASH score of 76.2 and Kretschmer *et al.* reported a score of 58 ± 26 in the completely injured patients. While Chaitanya Dodakundi^10^ *et al.* reported a mean DASH score of 36 ( ± 15), these results were in double free muscle transfer for the treatment of traumatic total BPI patients, which means those patients may have had better function recovery.

Traumatic global BPI results in a significant disability of function and psychosocial consequences. After a certain period of reconstructive surgery, the muscle power and ROM have been stabilized. It is hard to improve the outcome, even with complementary treatment measurements. Therefore, there is an increased need for evaluating the psychological status to improve the QOL of these patients. In this study, there were 45.24% and 42.86% of patients who suffered from depression and anxiety, respectively; while 35.71% of patients are suffering from both clinical anxiety and depression according to these scales. The current study showed a higher prevalence of clinical anxiety and clinical depression among the traumatic global BPI patients, in comparison with that in population (5–10%)^34^. Therefore, the mental health in traumatic global BPI patients should be given attention. Multidisciplinary treatment program involving mental health services are needed to help BPI patients to cope with these problems.

One of the limitations is this study lacks preoperative DASH, SDS and SAS scores of these patients. Another limitation is that although no statistically significance was found between patients with and without bowstring complications or donor site complications in elbow flexion and wrist extension muscle power or ROM; this might be resulted as the insufficient sample size.

## Conclusion

With a well-trained team, free gracilis transfer using an accessory nerve as a donor nerve is a satisfactory treatment to reconstruct the elbow flexion and wrist extension in global BPI patients.

Unsatisfactory outcomes caused by a certain degree of complications can be avoided by prompt and appropriate intervention.

Psychological intervention is needed because of the high prevalence of clinical anxiety and clinical depression.

## Additional Information

**How to cite this article**: Yang, Y. *et al.* Functioning free gracilis transfer to reconstruct elbow flexion and quality of life in global brachial plexus injured patients. *Sci. Rep.* **6**, 22479; doi: 10.1038/srep22479 (2016).

## Figures and Tables

**Table 1:** Demographic characteristics of patients (N = 42).

Variables	frequency/mean	Percentage/standard deviation
Gender		
Female	2	4.76
Male	40	95.24
Investigated arm		
Left	29	69.05
Right	13	30.95
Cause of injury		
Motorcycle	32	76.16
Automobile	2	4.76
Fall	5	11.90
Traction	1	2.38
Trauma	2	4.76
Age*	26.36	6.77
BMI*	22.21	3.59
Follow-up times**	54.5	25.5 ~ 83.5

^*^Data were presented as mean ± standard deviation.

^**^Data were presented as median and quartile range.

**Table 2:** Muscle power and ROM of elbow flexion and wrist extension.

Variables	Frequency/Median	Percentage/Quartile range
Muscle power		
Elbow flexion		
poor	5	11.90
fair	1	2.38
good	15	35.71
excellent	21	50.00
Wrist extension		
poor	15	35.71
fair	18	42.86
good	6	14.29
excellent	3	7.14
ROM*		
Elbow flexion	106.50	80 ~ 115.75
Wrist extension	17.00	0 ~ 25.25
TAFE	26.00	6.50 ~ 48.50

^*^Data were presented as median and quartile range ROM = range of motion TAFE = total active fingers extension.

**Table 3:** Quality of life characteristics of patients (N = 42).

Variable	Frequency	Percentage
NRS		
0	9	21.43
1–3	12	28.57
4–6	13	30.95
7–10	8	19.05
SDS		
<53	23	54.76
53 ~	7	16.67
62 ~	8	19.05
≫72	4	9.52
SAS		
<50	24	57.14
50 ~	9	21.43
60 ~	8	19.05
≫70	1	2.38
DASH*	51.14	20.97

SDS = Self-Rating Depression Scale SAS = Self-Rating Anxiety Scale.

DASH = Disabilities of the arm shoulder and hand.

^*^Data were presented as mean ± standard deviation NRS = Numeric Rating Scale.

**Table 4:** Comparison of muscle power between different groups.

Muscle power	poor	fair	good	excellent	*Z/χ2*	*P*
Elbow flexion						
Age group					0.487	0.626
10 ~ 30	5	1	10	16		
31 ~ 50	0	0	5	5		
BMI					5.402*	0.067
<18.5	2	0	4	1		
18.5 ~ 24.9	2	0	8	16		
≫25	1	1	3	4		
Hand					1.121	0.262
Left	3	0	10	16		
Right	2	1	5	5		
Follow-up times					1.493	0.135
≪2 years	2	0	4	4		
>2years	3	1	11	17		
Complications					0.983	0.326
Without	4	1	14	16		
With	1	0	1	5		
Bowstring					0.422	0.673
Without	5	1	11	17		
With	0	0	4	4		
Wrist extension						
Age group					0.806	0.42
10 ~ 30	12	14	5	1		
31 ~ 50	3	4	1	2		
BMI					1.099*	0.577
<18.5	3	4	0	0		
18.5 ~ 24.9	9	10	4	3		
≫25	3	4	2	0		
Hand					0.743	0.458
Left	12	11	3	3		
Right	3	7	3	0		
Follow-up time					1.465	0.143
≪2 years	4	6	0	0		
>2years	11	12	6	3		
Complications					0.596	0.551
Without	13	15	5	2		
With	2	3	1	1		
Bowstring					0.103	0.918
Without	13	13	5	3		
With	2	5	1	0		

^*^Data were presented as χ2 BMI = Body Mass Index.

**Table 5:** Comparison of ROM between different groups.

ROM		Z/χ2	P
Elbow flexion			
Age group		0.03	0.976
10 ~ 30	109(60.5,118.75)		
31 ~ 50	105(87.5,115.75)		
BMI		0.168*	0.919
<18.5	110(0,136)		
18.5 ~ 24.9	106.5(91.5,115)		
≫25	98(60,121.5)		
Hand		0.273	0.785
Left	110(80,115)		
Right	94(75,121)		
Follow-up time		0.811	0.417
≪2 years	93(45,110)		
>2years	110(90.5,121.5)		
Complications		0.237	0.813
Without	105(80,118)		
With	110(60,110)		
Bowstring		0.675	0.5
Without	107.5(61.5,115.75)		
With	102(92.5,126.25)		
Wrist extension			
Age group		0.768	0.443
10 ~ 30	17(0,25)		
31 ~ 50	17.5(4.5,36.25)		
BMI		1.947*	0.378
<18.5	8(0,19)		
18.5 ~ 24.9	20(0,26.25)		
≫25	10(2.5,36.5)		
Hand		0.61	0.542
Left	10(0,25.5)		
Right	20(5,31)		
Follow-up time		1.138	0.255
≪2 years	6.5(0,29.75)		
>2years	19.5(0,25)		
Complications		1.497	0.134
Without	15(0,24)		
With	25(0,48)		
Bowstring		0.212	0.832
Without	14.5(0,26.25)		
With	17.5(3.75,24.25)		

^*^Data were presented as χ2 ROM = range of motion BMI = Body Mass Index.

**Table 6:** Complications of the free gracilis transfer.

Complications	Recipient site	Donor site
Short-term		
thrombosis		
arterial thrombosis	2	0
venous thrombosis	2	0
partial necrosis		
proximal	1	0
distal	2	0
fat liquefaction	2	0
hematoma	1	2
numbness	0	5
reduction of adduction strength	0	1
Long-term		
bowstringing	8	0
elbow stiffness	1	0
numbness	0	3
hypoesthesia	0	1
knee instability	0	1
mild edema	0	1
